# Biomass Allocation Patterns across China’s Terrestrial Biomes

**DOI:** 10.1371/journal.pone.0093566

**Published:** 2014-04-07

**Authors:** Limei Wang, Longhui Li, Xi Chen, Xin Tian, Xiaoke Wang, Geping Luo

**Affiliations:** 1 State Key Laboratory of Desert and Oasis Ecology, Xinjiang Institute of Ecology and Geography, Chinese Academy of Sciences, Urumqi, China; 2 Institute of Forest Information Resource Techniques, Chinese Academy of Forestry, Beijing, China; 3 Beijing Urban Ecosystem Research Station, State Key Lab of Urban and Regional Ecology Research Center for Eco-Environmental Sciences, Chinese Academy of Sciences, Beijing, China; Lakehead University, Canada

## Abstract

Root to shoot ratio (RS) is commonly used to describe the biomass allocation between below- and aboveground parts of plants. Determining the key factors influencing RS and interpreting the relationship between RS and environmental factors is important for biological and ecological research. In this study, we compiled 2088 pairs of root and shoot biomass data across China’s terrestrial biomes to examine variations in the RS and its responses to biotic and abiotic factors including vegetation type, soil texture, climatic variables, and stand age. The median value of RS (RSm) for grasslands, shrublands, and forests was 6.0, 0.73, and 0.23, respectively. The range of RS was considerably wide for each vegetation type. RS values for all three major vegetation types were found to be significantly correlated to mean annual precipitation (MAP) and potential water deficit index (PWDI). Mean annual temperature (MAT) also significantly affect the RS for forests and grasslands. Soil texture and forest origin altered the response of RS to climatic factors as well. An allometric formula could be used to well quantify the relationship between aboveground and belowground biomass, although each vegetation type had its own inherent allometric relationship.

## Introduction

Belowground biomass (BGB) is an important component of global terrestrial ecosystem carbon stocks and plays a critical role in global carbon cycling. Belowground biomass is more difficult and costly to measure and is a major source of uncertainties in large-scale biomass estimation and global carbon cycles [1]–[3]. The partitioning between aboveground and belowground biomass influences many of the functions performed by diverse terrestrial communities as well as the functions performed by individual plants (e.g., [4]–[7]). Root to shoot ratio (RS) is an effective parameter to describe the allocation between aboveground biomass (AGB) and BGB, and thus provides a practical tool to estimate BGB by relatively easily measured AGB. The RS reflects a plant’s specific adaptive responses to its environment and has been widely used as a key descriptor for terrestrial ecosystem carbon modeling [8].

Biomass partitioning between belowground and aboveground parts can be predicted by plant allometric relationships [6], [9]. At the level of individual plants, AGB scales nearly isometrically with respect to BGB, and this relationship has been validated across a broad spectrum of ecologically diverse vascular plants spanning several orders of magnitude in total body mass [10]. The allometric theory also estimates an isometric relationship between AGB and BGB at the community level [11], which has not been examined by adequate field measurements, especially its generality across diverse vegetation groups.

Recent studies have indicated that RS changes with environmental factors (e.g., climate, CO_2_, soil texture, soil moisture, and nitrogen), biotic factors (e.g., plant type, stand age, and leaf traits), and forest origins [1], [12]–[15]. Some improvements have been made in determining biomass allocation; however, many of the past studies focused on a particular ecosystem, and there have been few comprehensive investigations of RS across multiple ecosystems. Investigating biomass allocation patterns and their relationships with environmental factors crossing multiple ecosystems at regional scale may shed new light on this important topic.

China has rich vegetation resources and diverse vegetation types. China has the fifth highest total forest area and second highest total grassland area among all countries in the world, and thus the country plays an important role in global carbon stocks and cycling. The numerous vegetation types, diverse soil textures, complicated land use/cover patterns, and varying climatic zones offer a unique chance to examine the variation of RS and its influencing factors. Validating RS data from diverse biomass also provides a good opportunity to test the allometric relationships for ecosystems partitioning between AGB and BGB. Considerable work has been conducted to investigate the performance of allometric theory for a specific ecosystem and at sub-regional scales. For example, Wang et al. [16] examined RS relationships for major forest types in northeast China and pointed out that they differed significantly between natural and planted forests, and between broadleaf and coniferous forests. Yang et al. [17] focused their investigation on the Tibetan grasslands and suggested that the isometric relationships between AGB and BGB did not differ significantly between alpine steppe and alpine meadow. Further, Yang et al. [17] investigated relationships between AGB and BGB of grassland ecosystems across northern China and indicated that AGB was nearly proportional to BGB with a scaling exponent across various grassland types at the community level with no significant difference between temperate and alpine grasslands or between steppe and meadow. Comprehensive research on biomass allocation and its response to both climate and soil types for multiple ecosystems are essential to improving our understanding of the general rules of biomass allocation for biomes.

In this study, we compiled a comprehensive database of biomass and RS for the major terrestrial biomes across China. We used statistical approaches to address: 1) how RS varies across China’s terrestrial biomes, 2) how RS responds to biotic and abiotic variables, and 3) how well the allometric theory performs in comparison with estimates of the empirical relationship between RS and environmental variables.

## Materials and Methods

### Datasets

**Figure 1:**
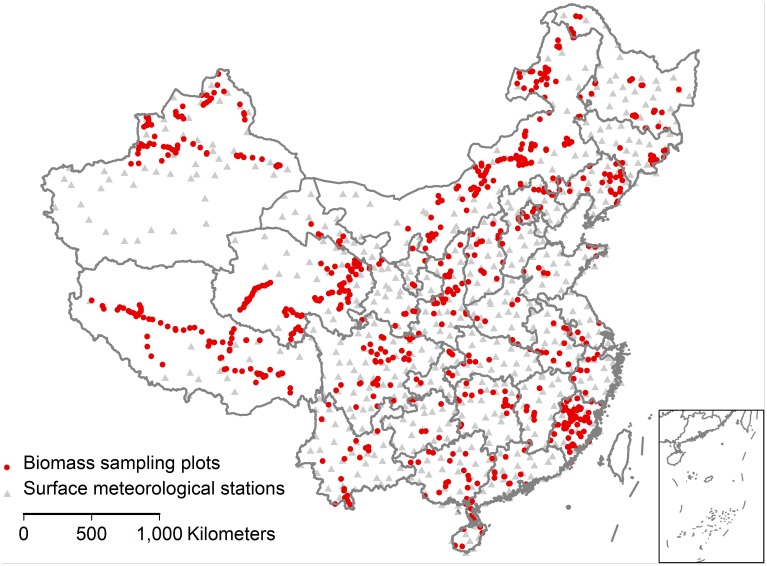
The geographical distribution of biomass sampling plots collected in this study. These data including forests, shrublands, and grasslands distributed across China.

We collected 2088 measurements of root and shoot biomass across China from hundreds of published papers (Fig. 1, Appendix S1). In the database, 276 records were extracted from Wang et al. [16], 377 records were extracted from Yang et al. [8], and 1139 records were extracted from Luo et al. [18]. The rest of the records in the dataset were compiled by the authors from related studies.

For each data set, we recorded the biomass data and whatever supporting information was available, including (1) shoot, root, and total biomass (dry weight of biomass per unit area); (2) site descriptions (including location, latitude, longitude, elevation, mean annual temperature, mean annual precipitation, mean annual potential evapotranspiration (PET), and soil texture); (3) vegetation descriptions (including vegetation type, dominate species, forest origin (primary, secondary, planted forest), age, height, density of stems, and mean diameter at breast height (DBH)); (4) descriptions of shoot and root biomass sampling methods (including sample size, dimensions of soil cores or soil pits, depth of sampling, whether root crowns were sampled, and whether existing allometric theories were applied).

The climatic variables mean annual precipitation (MAP) and temperature (MAT) at each biomass-sampling site were determined from the nearest surface meteorological stations across China (Fig. 1). Historically mean values of MAP and MAT during the period from 1961 to 2010 were used for analysis. Among 2088 sites, 77 sites have attached MAP and 88 sites have attached MAT. We assessed the accuracy of extracted MAP and MAT by comparing with site-attached values and found that extracted MAP was significantly correlated with the recorded measurements (*r* ^2^ = 0.92, n = 77, *p*<0.01), and extracted MAT was in good agreement with the measurements (*r* ^2^ = 0.9, n = 88, *p*<0.01) as well. We computed mean annual potential evapotranspiration (PET) using the FAO Penman–Monteith equation [19] from the meteorological variables recorded by the 664 surface stations, and then we computed potential water deficit index (PWDI) by the ratio of the mean annual precipitation to annual potential evapotranspiration (MAP/PET) [20].

### Vegetation Types

The categorization of data into vegetation types relied primarily upon a soil texture classification scheme [17], [18]. Different vegetation types had obviously different RS and soil texture may alter the value of RS for same vegetation types. Based on a soil texture map (Institute of Soil Science, Chinese Academy of Sciences, 1986), the dataset was divided into eight types: loam forest, sandy forest, clay forest, loam grassland, sandy grassland, clay grassland, loam shrubland, and sandy shrubland (Table 1). Recent studies have indicated that RS shows different physiological and ecological responses to varied surroundings for a range of forest stand age and climate zones [8], [12], [16]. Summarizing these studies, the forests and grasslands were further investigated based on five groups divided by the forest origin and climatic zone: primary forest, secondary forest, planted forest, temperate grassland, and alpine grassland (Table 1).

**Table 1:** Median of above–ground biomass (AGB), below–ground biomass (BGB), root to shoot ratio (RS) and allometric relationships between AGB and BGB (*p*<0.05) across China’s biomes.

Vegetation category	AGB(Mg ha^−1^)	BGB(Mg ha^−1^)	RS	Range	n	LogAGB–logBGB models		
						Slope	Intercept	*r* ^2^
Vegetation type								
Primary forest	99.1	24.0	0.24	0.03–1.20	454	0.87	−0.37	0.85
Secondary forest	93.7	22.6	0.23	0.08–0.58	97	0.95	−0.52	0.78
Planted forest	68.4	15.5	0.22	0.05–0.90	1002	0.81	−0.30	0.76
Temperate grassland	1.5	7.7	5.73	0.43–29.2	243	0.77	0.74	0.58
Alpine grassland	0.7	4.1	6.27	0.81–45.6	262	1.00	0.79	0.73
Soil texture								
Loam forest	76.5	16.7	0.22	0.02–1.20	967	0.90	−0.45	0.82
Sand forest	62.2	15.6	0.25	0.03–0.70	242	0.82	−0.30	0.79
Clay forest	91.1	19.3	0.22	0.05–0.50	345	0.87	−0.42	0.87
Loam grassland	1.0	5.5	6.10	0.43–29.2	336	0.78	0.74	0.66
Sand grassland	0.7	5.0	5.61	0.56–45.6	153	0.89	0.71	0.59
Clay grassland	1.1	7.2	7.60	2.0–12.72	10	0.96	0.87	0.74
Loam shrubland	9.5	11.3	0.71	0.26–1.56	12	1.00	−0.31	0.80
Sand shrubland	10.6	8.6	0.77	0.47–1.96	9	1.16	−0.44	0.95
Region								
China’s forests	76.7	17.0	0.23	0.03–1.20	1551	0.86	−0.36	0.81
Global forests	81.0	22.5	0.25	0.09–1.16	207	0.88	−0.32	0.90
China’s grasslands	1.0	5.2	6.00	0.43–45.6	502	0.82	0.74	0.63
Global grasslands	3.5	12.7	3.26	0.38–26.0	61	0.40	0.84	0.20
China’s shrublands	10.6	10.6	0.73	0.26–1.96	22	1.05	−0.16	0.89
Global shrublands	16.6	14.0	1.84	0.34–4.25	8	–	–	–

n, number of samples; Slope, the scaling exponent of the log–log linear functions; Intercept, the allometric constant of log–log linear functions; *r*, the coefficient of determination.

### Statistical Analysis

Descriptive analysis was used to determine the statistical characteristics (e.g., median value, range of data) of categorical data. Linear and nonlinear analyses were performed to develop regressions for RS dependent on the vegetation type, soil texture, and climatic variables. The significance of differences between slopes (scaling exponent) and y-intercepts (allometric constant) of the log-log transformed linear functions was evaluated by analyses of variance [21]. The standard error of the estimation (SEE) was used to measure the difference between predicted and actual values of root biomass.

## Results

### Variations of RS across China’s Biomes

The biomass and RS values shown in Table 1 are referred to as the median rather than the mean values for the skewed distribution of data for many of the vegetation types. RS values ranged from 0.03 to 1.2, 0.43 to 45.6, and 0.26 to 1.96 for forests, grasslands, and shrublands, respectively (Table 1). The median RS tended to decrease from grasslands to shrublands to forests across China (Table 1).

The median RS of forests tended to increase from planted to secondary to primary stands. In addition, forests growing in sandy soil had a larger median RS than those in loam and clay soils. Further, loam soil forests had approximately the same median RS value as the clay soil forests (Table 1).

For grasslands, RS changed significantly with different grassland types. The median RS of alpine grassland was much higher than that of temperate grassland (Table 1). There was a general trend for RS to vary with soil texture changes across grasslands. The median RS decreased from clay soil grassland to loam soil grassland to sandy soil grassland.

### Responses of RS to Climatic Variables

**Figure 2:**
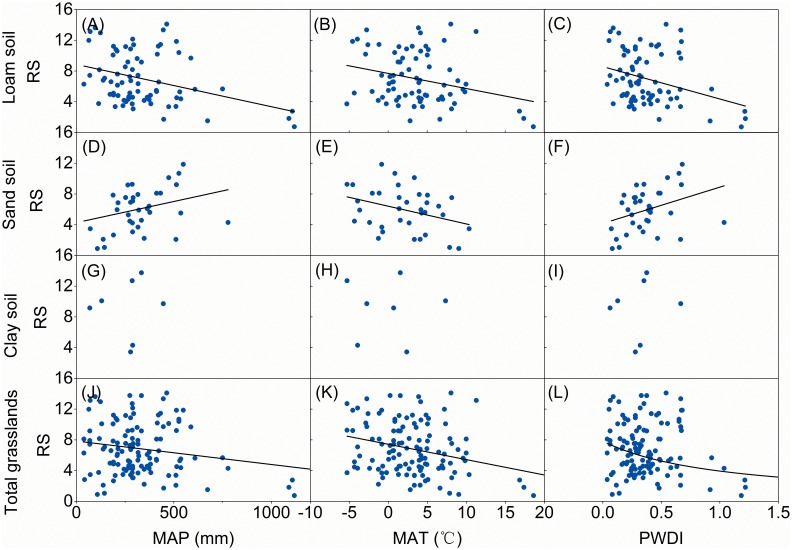
Root to shoot ratio (RS) for grasslands as a function of mean annual precipitation (MAP), temperature (MAT) and potential water deficit index (PWDI). Regression lines are given for loam, sandy, and claygrasslands and for all data together, if the relationships are significant at *p*<0.05.

**Figure 3:**
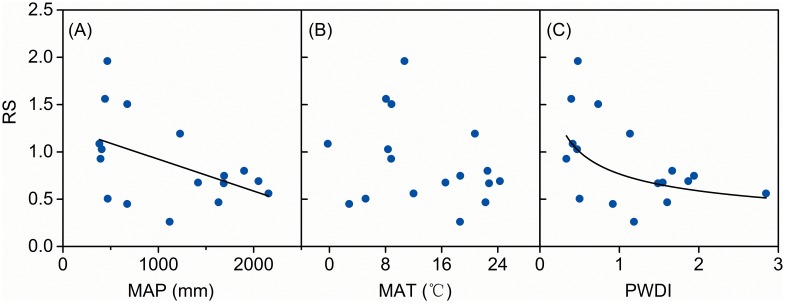
Root to shoot ratio (RS) for shrublands as a function of mean annual precipitation (MAP), temperature (MAT) and potential water deficit index (PWDI). Regression lines are given for all data together if the relationships are significant at *p*<0.05.

**Figure 4:**
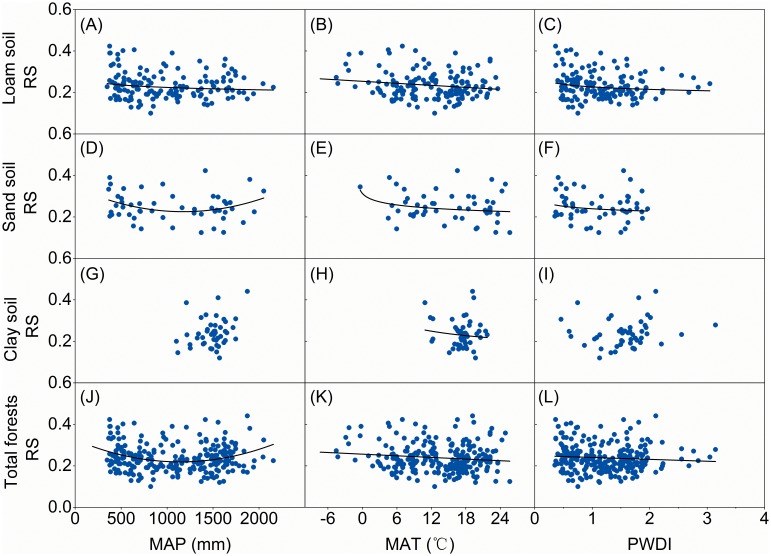
Root to shoot ratio (RS) for forests as a function of mean annual precipitation (MAP), temperature (MAT) and potential water deficit index (PWDI). Regression lines are given for loam, sandy, and clay forests and for all data together, if the relationships are significant at *p*<0.05.

China’s forests, grasslands and shrublands responded differently to climatic variables. For both grasslands (Fig. 2J) and shrublands (Fig. 3A), RS decreased significantly (*p*<0.01) with increased MAP. A similar trend was found in RS for forests where MAP was less than 1250 mm·yr^−1^; however, RS values tended to increase significantly (*p*<0.01) where MAP was over 1250 mm·yr^−1^ (Fig. 4J). Generally, soil nutrient availability dominated RS where precipitation was high (>1250 mm) [26] and more biomass was allocated into roots for uptaking nutrient and RS tend to increase with MAP. Although RS decreased significantly with increasing MAT for both forests (*p*<0.05, Fig. 4K) and grasslands (*p*<0.01, Fig. 2K), there was no significant relationship between MAT and RS for shrublands (Fig. 3B). RS values were negatively related to PWDI (*p*<0.01) for all forests (Fig. 4L), grasslands (Fig. 2L), and shrublands (Fig. 3C).

**Figure 5:**
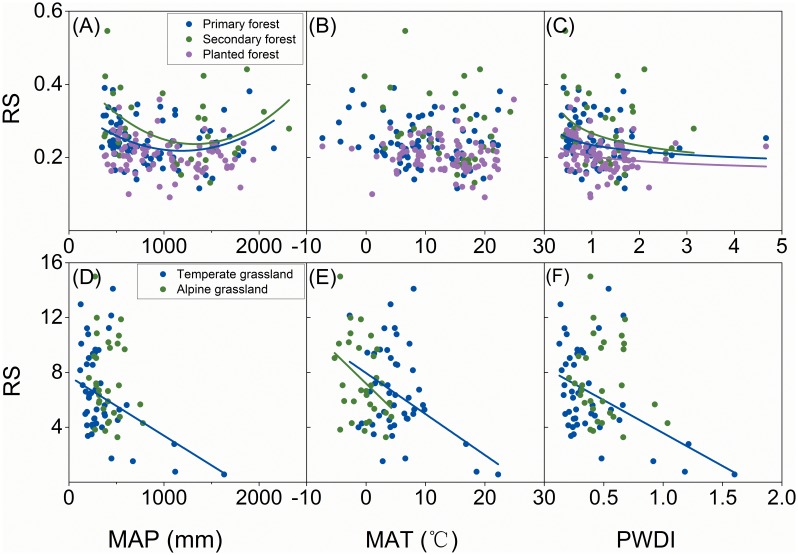
Root to shoot ratio (RS) as a function of mean annual precipitation (MAP), temperature (MAT) and potential water deficit index (PWDI): Forests (A–C) and Grasslands (D–F). Regression lines are given for every vegetation group if the relationships are significant at *p*<0.05.

Soil texture affected RS’s responses to climate. RS from both loam and sandy soils showed significant responses to climate variables for forests (Figs. 4A–F) and grasslands (Figs. 2A–F). However, no general trend was found between climate and RS from clay soil for either forests or grasslands (Figs. 2G–I). In addition to soil texture, the responses of RS to climate were influenced by forest origins. RS for natural forests showed a significant U-shaped trend (*p*<0.05) as MAP increased; however no trend existed between MAP and RS for planted forests (Fig. 5A). Neither natural nor planted forests showed a significant trend for RS as a function of MAT (Fig. 5B); however, RS decreased significantly (*p*<0.01) as PWDI increased for both natural and planted forests (Fig. 5C). Climatic zonation was an important factor influencing responses of grasslands’ RS to climate. RS for temperate grasslands was negatively related to MAP, MAT, and PWDI (Fig. 5D–F, *p*<0.01). In contrast, no significant trend existed between RS and MAP (Fig. 5D) or PWDI (Fig. 5F) for alpine grassland, although RS for alpine grassland decreased significantly (*p*<0.01) with increasing MAT (Fig. 5E).

### Allometric Relationships between Root and Shoot Biomass

**Figure 6:**
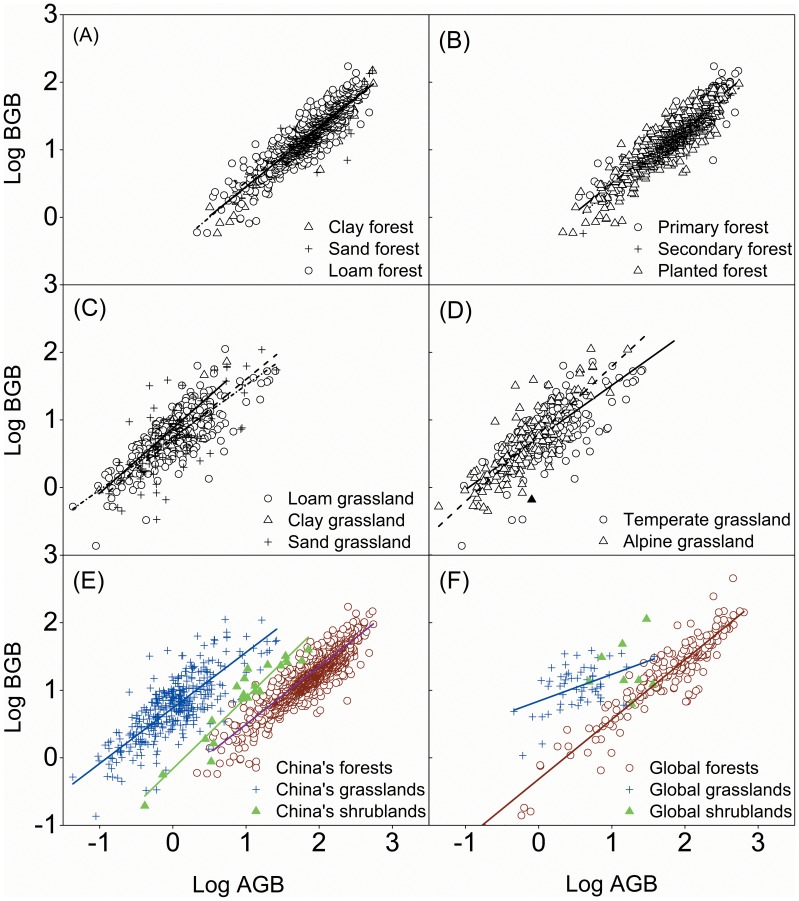
Allometric relationships between aboveground biomass (AGB) and belowground biomass (BGB) for different vegetation groups. (A) Comparison in between loam, sand and clay forest. (B) Comparison in between primary, secondary and planted forest. (C) Comparison in between loam, sand and clay grassland. (D) Comparison in between temperate and alpine grassland. (E) Comparison in between China’s forest, grassland and shrubland. (F) Comparison in between global forest, grassland and shrubland. Regression lines and equations are given for every vegetation group if the relationships are significant at *p*<0.05. Global biomass data are from Mokany et al. [12].

The relationships between root biomass (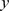) and shoot biomass (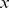) across China’s biomes fit well with an allometric power function, 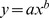 (Fig. 6). The coefficients for 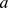 were 0.3, 8.02, and 2.35 and for 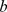 were 0.94, 0.72, and 0.66 for forests, grasslands, and shrublands, with corresponding 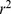 of 0.75, 0.5, and 0.85, respectively. In general, the allometric relationships between root and shoot biomass were significantly (*p*<0.01) different from each other between China’s major terrestrial ecosystems (forests, grasslands, and shrublands) and all slopes were significant (*p*<0.01) (Fig. 6; Table 1).

Across different forest types, none of the regressions between root and shoot biomass were significantly (*p*<0.05) different from each other and all slopes were significant (*p*<0.01) (Figs. 6A, B; Table 1). However, soil texture and climatic zonation altered grasslands’ allometric relationships. The slope of the regression for clay grasslands was significantly (*p*<0.01) greater than that of both loam and sandy grasslands. The allometric relationship for loam soil is not significantly different from that of sandy soil (*p*<0.01) (Fig. 6C; Table 1). The slope for alpine grasslands was significantly greater (*p*<0.01) than that of temperate grasslands. The allometric relationship for alpine grasslands was significantly different from that of temperate grasslands (*p*<0.01) (Fig. 6D; Table 1). Meanwhile, the allometric relationship for China’s grasslands was significantly different from the global average; however, the slope of the allometric equation for China’s forests was similar to the global value (Figs. 6E, F).

## Discussion

### Variations in RS across China’s Biomes

We reported that the overall median values of AGB were 76.7, 1.0 and 10.6, and the median of BGB were 17.0, 5.2 and 9.4 for China’s forests, grasslands and shrublands, respectively (Table 2). As a consequence, the resulted median RS were 0.23, 0.60 and 0.74 for China’s forests, grasslands and shrublands, respectively. Our estimated AGB, BGB and RS for grasslands were quite comparable with previous research using Northern China’s dataset [8]. However, a remarkable difference existed in terrestrial root and shoot biomass and RS between China and global averages. Both root and shoot biomass in China were lower than global values for each vegetation type (Table 3). The median RS values for China’s forests (0.23) and shrublands (0.73) were lower than global estimates (0.29 and 2.45, respectively [12]); however, the median RS for China’s grasslands (6.0) was much higher than global estimates (3.3 [12] and 3.7 [22]). Such difference may come from several aspects. Firstly, sampling methods and the number of dataset used may be one of the main reasons. Makony et al. [24] pointed that data omitting was critical to the analysis of root and shoot ratio. Limited by axillary information for the dataset used in the current research, we ignored the selection and keep all data points we collected in analysis. Such treatment may avoid distorting the possible nature of biomass allocation pattern across wide biome, climate and soil conditions. Secondly, accuracy of climatic variables (mean annual precipitation and temperature) and soil properties (soil texture) may contribute the uncertainties of estimated RS values. Particularly for those data sites located in different altitude or slope of mountain areas, different climatic conditions should be expected but same climatic variables were used for analysis due to the lack of available meteorological observations. To overcome this drawback, we averaged all data points where shared meteorological measurements. However, the detailed response of RS to climatic variables may be also masked.

**Table 2:** Statistical analysis for predicted root biomass (BGB) using median vegetation-specific root to shoot ratio (RS) and allometric equations.

Vegetation category		Predicted root biomass by median vegetation–specific RS			Predicted root biomass by allometric equations		
		Slope	*r* ^2^	SEE	Slope	*r* ^2^	SEE
Vegetation type							
Primary forest	443	0.95	0.77	12.1	1.09	0.76	12.1
Secondary forest	97	1.00	0.68	11.1	1.10	0.68	11.0
Planted forest	997	0.72	0.70	7.24	0.76	0.71	7.22
Temperate grassland	217	1.42	0.79	30.2	2.66	0.66	38.1
Alpine grassland	258	1.36	0.76	12.7	2.22	0.68	14.7
Soil texture							
Loam forest	951	0.97	0.72	9.68	1.01	0.72	9.66
Sand forest	242	0.82	0.78	8.50	0.97	0.78	8.63
Clay forest	342	0.89	0.81	7.85	0.93	0.80	7.92
Loam grassland	312	0.54	0.42	12.5	0.96	0.45	12.1
Sand grassland	152	0.73	0.56	11.8	1.18	0.60	11.3
Clay grassland	12	0.32	0.008	5.18	0.51	0.08	4.99
Loam shrubland	11	0.73	0.84	5.30	1.03	0.89	4.50
Sand shrubland	9	1.35	0.66	4.85	1.26	0.65	4.95
Region							
China’s forests	1535	0.90	0.75	9.15	0.98	0.74	9.16
Global forests	207	1.02	0.56	30.1	0.98	0.56	30.1
China’s grasslands	475	0.60	0.46	12.4	1.05	0.50	12.0
Global grasslands	57	0.13	0.04	9.66	0.71	0.04	9.66
China’ s shrublands	21	0.76	0.79	5.26	1.04	0.85	4.53
Global shrublands	8	–	–	–	–	–	–

n is number of samples, Slope is the scaling exponent of the regressions, *r* is coefficient of determination and SEE is standard error of the estimation.

**Table 3:** Above–ground biomass (AGB), below–ground biomass (BGB) and root to shoot ratio (RS) for forests, grasslands and shrublands from different studies.

Vegetation category	AGB (Mg ha^−1^)	BGB (Mg ha^−1^)	RS	Reference
Forest				
China’s forests	76.7	17.0	0.23	This study
China’s forests	–	–	0.23	Luo et al. (2012)
Global forests	81.0	22.5	0.25	Mokany et al. (2006)
Grassland				
China grasslands	1.0	5.2	6.0	This study
China grasslands	1.0	5.7	5.7	Yang et al. (2009)
Global grasslands	3.5	12.7	3.3	Mokany et al. (2006)
Global grasslands	3.8	14.0	3.7	Jackson et al. (1996)
Shrubland				
China’s shrublands	10.6	9.7	0.73	This study
Global shrublands	16.6	14.0	1.84	Mokany et al. (2006)

Arithmetic mean or median values are presented to compare with other studies.

### Factors Influencing Biomass Allocation

Biomass allocation is one of the most successful theories in modern ecology [36]. Although plant growth and biomass allocation are highly influenced by environmental and non-environmental factors, a mass number of previous research have demonstrated that biomass allocation is impacted by climatic variables (particularly precipitation and temperature), soil type, nutrient availability and plant species [17], [23]–[26], [32], [33].

Majority of previous studies reported that RS can be either positively or negatively and linearly correlated and even nonlinearly related with the increase in annual precipitation at relatively larger regional or national scales [8], [16], [37], [38], [18], [28] but the value of RS overall decreased with precipitation at global scale [3]. Similarly, the effect of temperature on RS can be different from region to region, vegetation type to type [18], [38], [27]. Various possible combinations of hydrothermal conditions (precipitation and temperature) may explain the variation of the diverse response of RS to individual factor of precipitation and temperature. Further, soil texture, vegetation type, composition and age, nutrient availability also induced variations in the RS across ecosystems [3]. Generally, there was an increasing trend for RS as soil texture changed from clay (0.22) to sand (0.25) for forests (Table 1). Lower water and nutrient availability from sandy soil may be responsible for the larger RS. In contrast, RS values of grasslands showed trend of decreasing when soil texture changed from clay to sand (Table 1). Our results also found that RS is more sensitive to MAT than MAP in grasslands. Lower temperatures in clay soil caused roots to gain more biomass to maintain energy and acquire water and nutrients [21], [31], which may be responsible for the higher RS for clay grasslands.

One of key contributions of this study is that we comprehensively examined the responses of RS to multiply abiotic and biotic factors, including climatic variables (MAP, MAT, and PWDI), soil texture and vegetation type and age. The integrated analysis provided comprehensive understanding on the variations of RS and their response of RS to environmental factors. We found that RS could be significantly impacted by any of considered abiotic or biotic factors. Compared with any single factor from climate, a comprehensive index PWDI could be better to describe the dependency of RS on environmental factors. Biomass partition is commonly viewed as a result impacted by multiple environmental factors. Variation in RS and difference in their responses to individual or combined environmental factors supported the optimal partitioning hypothesis [29], [30], suggesting an adaptation for plants in varied environments. It is widely recognized that RS varies with various biotic (e.g., stand age, vegetation type, and growing period) and abiotic factors (e.g., soil texture, soil nutrients, climate, and plant origin), as demonstrated in our analysis, although the mechanism is not well understood and inconsistencies between findings from existing studies are still quite large.

Differing from the analysis between RS and environmental factors, allometric equations have been also widely accepted as an effective method for investigating biomass partitioning between aboveground and belowground components (e.g., [1], [34], [35]). As an alternative, we attempt to examine whether the vegetation-specific RS presented in this study would provide a more accurate and easier means for estimating root biomass than allometric equations. We used the root and shoot biomass data collected in this study to compare the predictive accuracy of the two methods. We found that the application of median RS predicted root biomass with approximately the same accuracy as the allometric equations for 7 of the 13 vegetation groups (Table 2). Among the other 6 groups, we had a better prediction of root biomass using median RS for temperate and alpine grasslands, but lower predictive accuracy in loam, sandy, and clay grasslands and loam shrublands (Table 2). Moreover, the application of vegetation-specific RS for forests across soil textures had better predictive accuracy for root biomass than the single median RS for total forests in China. Similarly, the vegetation-specific RS for temperate and alpine grasslands also had better predictive accuracy for root biomass than the single median RS for total grasslands in China. This indicated that the accuracy of root biomass prediction varies significantly across vegetation types, particularly for grasslands, emphasizing that it is essential to advance our understanding of RS dynamics across vegetation types for improving the accuracy of root biomass predictions [12].

## Conclusions

This paper systematically examined the variation in RS across China’s terrestrial biomes and its influencing factors including vegetation type, soil properties, climatic variables, and stand age. We found that median RS tended to decrease from grasslands (6.0) to shrublands (0.73) to forests (0.23) in China. Although primarily decided by the plants’ inherent isometric relationships, RS were significantly (*p*<0.05) influenced by vegetation type, soil texture, climatic variables, and stand age.

RS showed general trends with changing mean annual temperature, mean annual precipitation, and potential water deficit index. RS values were negatively related to mean annual precipitation and positively related to potential water deficit for forests, grasslands, and shrublands. For both forests and grasslands, RS were negatively related to mean annual temperature. Soil texture, forest origin, and climatic conditions affected the size of the RS and its relationships with climatic factors. Allometric equations performed better than empirical vegetation-specific RS equations in 6 out of 13 vegetation types.

## Supporting Information

Appendix S1 Dataset on the root to shoot ratio, soil and vegetation types and climatic variables across China’s terrestrial biomes.
(XLSX)

## References

[1] Cairns MA, Brown S, Helmer EH, Baumgardner GA (1997) Root biomass allocation in the world’s upland forests. Oecologia 111: 1–11. 28307494 10.1007/s004420050201

[2] Reich PB (2002) Root–shoot relations: optimality in acclimation and adaptation or the ‘‘Emperor’s New Clothes’’? In: Waisel Y, Eshel A, Kafkafi U, editors. Plant roots: The hidden half. Basel, Switzerland: Marcel Dekker. 205–220.

[3] Mokany K, Raison RJ, Prokushkin AS (2005) Critical analysis of root:shoot ratios in terrestrial biomes. Glob Chang Biol 11: 1–13.

[4] Poorter H, Nagel O (2000) The role of biomass allocation in the growth response of plants to different levels of light, CO_2_, nutrients and water: A quantitative review. Aust J Plant Physiol 27: 595–607.

[5] Binkley D, Stape JL, Ryan M (2004) Thinking about resource use efficiency in forests. For Ecol Manage 193: 5–16.

[6] Niklas KJ (2005) Modeling below- and above-ground biomass for non-woody and woody plants. Ann Bot 95: 315–321. 15546927 10.1093/aob/mci028 PMC4246831

[7] Hui DF, Jackson RB (2006) Geographical and interannual variability in biomass partitioning in grassland ecosystems: a synthesis of field data. New Phytol 169: 85–93. 16390421 10.1111/j.1469-8137.2005.01569.x

[8] Yang YH, Fang JY, Ma WH, Guo DL, Mohammat A (2010) Large-scale pattern of biomass partitioning across China’s grasslands. Global Ecol Biogeogr 19: 268–277.

[9] Enquist BJ, Niklas KJ (2002) Global allocation rules for patterns of biomass partitioning across seed plants. Science 295: 1517–1520. 11859193 10.1126/science.1066360

[10] Niklas KJ (2006) A phyletic perspective on the allometry of plant biomass-partitioning patterns and functionally equivalent organ-categories. New Phytol 171: 27–40. 16771980 10.1111/j.1469-8137.2006.01760.x

[11] Cheng DL, Niklas KJ (2007) Above- and below-ground biomass relationships across 1534 forested communities. Ann Bot 99: 95–102. 17085476 10.1093/aob/mcl206 PMC2802968

[12] Mokany K, Raison RJ, Prokushkin AS (2006) Critical analysis of root:shoot ratios in terrestrial biomes. Glob Chang Biol 12: 84–96.

[13] Li LH, Yu Q, Zheng YF, Wang J, Fang QX (2006) Simulating the response of photosynthate partitioning during vegetative growth of winter wheat to environmental factors. Field Crops Res 96: 133–141.

[14] Camila AC, Henrik S, Linda G, Annika N, Ulrika G, et al (2011) Patterns of plant biomass partitioning depend on nitrogen source. PLoS One 6: e19211. 21544211 10.1371/journal.pone.0019211 PMC3081341

[15] Yu Q, Wu H, He N, Lü X, Wang Z, et al (2012) Testing the growth rate hypothesis in vascular plants with above- and below-ground biomass. PLoS One 7: e32162. 22427823 10.1371/journal.pone.0032162 PMC3302800

[16] Wang XP, Fang JY, Zhu B (2008) Forest biomass and root–shoot allocation in northeast China. For Ecol Manage 255: 4007–4020.

[17] Yang YH, Fang JY, Ji CJ, Han WX (2009) Above- and belowground biomass allocation in Tibetan grasslands. Journal of Vegetation Science 20: 177–184.

[18] Luo YJ, Wang XK, Zhang XQ, Booth TH, Lu F (2012) Root:shoot ratios across China’s forests: Forest type and climatic effects. For Ecol Manage 269: 19–25.

[19] FAO (1998) Crop evapotranspiration–Guidelines for computing crop water requirements. Rome, Italy: Food and Agriculture Organization of the United Nations.

[20] Brown S, Lugo AE (1982) The storage and production of organic matter in tropical forests and their role in the global carbon cycle. Biotropica 14: 161–187.

[21] Crawley MJ (2002) Statistical computing–An introduction to data analysis using S-Plus. New York: John Wiley & Sons.

[22] Jackson RB, Canadell J, Ehleringer JR, Mooney HA, Sala OE, et al (1996) A global analysis of root distributions for terrestrial biomes. Oecologia 108: 389–411. 28307854 10.1007/BF00333714

[23] Ericsson T, Rytter L, Vapaavuori E (1996) Physiology of carbon allocation in trees. Biomass Bioenerg 11: 115–127.

[24] Matsui Y, Fukuda Y, Inoue T, Matsushita T (2003) Effect of natural organic matter on powdered activated carbon adsorption of trace contaminants characteristics and mechanism of competitive adsorption. Water Res 37: 4413–4424. 14511712 10.1016/S0043-1354(03)00423-8

[25] Gholz HL, Vogel SA, Cropper WP Jr, McKelvey K, Ewel KC, et al (1991) Dynamics of canopy structure and light interception in *Pinus elliottii* stands, north Florida. Ecol Monogr 61: 35–51.

[26] Agren GI, Franklin O (2003) Root: shoot ratios, optimization and nitrogen productivity. Ann Bot 92: 795–800. 14565938 10.1093/aob/mcg203 PMC4243620

[27] Read JJ, Morgan JA (1996) Growth and partitioning in *Pascopyrum smithii* (C3) and *Bouteloua gracilis* (C4) as influenced by carbon dioxide and temperature. Ann Bot 77: 487–496.

[28] Li LH, Wang YP, Yu Q, Pak B, Eamus D, et al (2012) Improving the responses of the Australian community land surface model (CABLE) to seasonal drought. J Geophys Res 117: G04002.

[29] Friedlingstein P, Joel G, Field CB, Fung IY (1999) Toward an allocation scheme for global terrestrial carbon models. Glob Chang Biol 5: 755–770.

[30] McConnaughay KDM, Coleman JS (1999) Biomass allocation in plants: ontogeny or optimality? A test along three resource gradients. Ecology 80: 2581–2593.

[31] Gower ST, Vogt KA, Grier CC (1992) Carbon dynamics of Rocky Mountain Douglas scheme-fir: Influence of water and nutrient availability. Ecol Monogr 62: 43–65.

[32] Schenk HJ, Jackson RB (2002) Rooting depths, lateral root spreads and below-ground/above-ground allometries of plants in water-limited ecosystems. J Ecol 90: 480–494.

[33] Kellomäki S, Wang KY (2001) Photosynthetic responses to needle water potentials in Scots pine after a four-year exposure to elevated CO_2_ and temperature. Tree Physiol 16: 765–772. 10.1093/treephys/16.9.765 14871683

[34] Vogt KA, Vogt DJ, Bloomfield J (1998) Analysis of some direct and indirect methods for estimating root biomass and production of forests at an ecosystem level. Plant Soil 200: 71–89.

[35] Snowdon P, Eamus D, Gibbons P, Khanna PK, Keith H, et al. (2000) Synthesis of allometrics, review of root biomass and design of future woody biomass sampling strategies. NCAS Technical Report 17. Canberra: Australian Greenhouse Office.

[36] Stearns SC (1992) The evolution of life histories. Oxford: Oxford University Press.

[37] Kang M, Dai C, Ji W, Jiang Y, Yuan Z, et al (2013) Biomass and its allocation in relation to temperature, precipitation, and soil nutrients in Inner Mongolia grasslands, China. PloS One 8(7): e69561 doi:10.1371/journal.pone.0069561 23936045 10.1371/journal.pone.0069561 PMC3723834

[38] Luo YJ, Wang XK, Zhang XQ, Ren Y, Poorter H (2013) Variation in biomass expansion factors to forest type, climate and stand development. Annals of Forest Sciences 70: 589–599.

